# Chinese Herbal Medicine Improves the Long-Term Survival Rate of Patients With Chronic Kidney Disease in Taiwan: A Nationwide Retrospective Population-Based Cohort Study

**DOI:** 10.3389/fphar.2018.01117

**Published:** 2018-10-01

**Authors:** Kuo-Chin Huang, Yuan-Chih Su, Mao-Feng Sun, Sheng-Teng Huang

**Affiliations:** ^1^Department of Chinese Medicine, China Medical University Hospital, Taichung, Taiwan; ^2^School of Chinese Medicine, China Medical University, Taichung, Taiwan; ^3^Graduate Institute of Integrated Medicine, College of Chinese Medicine, China Medical University, Taichung, Taiwan; ^4^Management Office for Health Data, China Medical University Hospital, Taichung, Taiwan; ^5^Department of Medical Research, Cancer Research Center for Traditional Chinese Medicine, China Medical University Hospital, Taichung, Taiwan; ^6^Chinese Medicine Research Center, China Medical University, Taichung, Taiwan; ^7^Research Center for Chinese Herbal Medicine, China Medical University, Taichung, Taiwan

**Keywords:** chinese herbal medicine, chronic kidney diseases, national health insurance research database, retrospective cohort study, survival rate

## Abstract

**Background and purpose:** Chinese herbal medicine (CHM) is frequently applied in conjunction with western pharmacotherapy to relieve symptoms in patients with CKD. However, evidence-based research into the effectiveness of CHM use as applied to treat CKD is limited and warrants further investigation. The aim of this study is to assess whether adjunctive treatment with CHM affected survival rate of CKD patients undergoing conventional western medical management.

**Methods:** A total of 14,718 CKD patients, including 6,958 CHM users and 7,760 non-CHM users, were recruited from the Longitudinal Health Insurance Database 2000, a sub-dataset of the National Health Insurance Research Database, to conduct this study. Demographic characteristics, including sex, age, job type, residential area, and comorbidity were considered as covariates to adjust the analysis. A network analysis of treatments, including with herbal formulas and single herbs, was performed to investigate the core patterns of CHM use for the treatment of CKD patients. The Kaplan-Meier method was used to determine the survival rate between CHM and non-CHM groups.

**Results:** After matching for sex and age, there were 550 subjects in both the CHM and non-CHM cohorts. Other than presence of diabetes (adjusted *OR* = 0.57, *p* < 0.001) and urinary tract infection (adjusted OR = 0.69, *p* < 0.05), sex, age, job type, area of residence, and other comorbidities indicated no special preference for CHM use among subjects. *Salvia miltiorrhiza* Bunge (SM) and Ji-Sheng-Shen-Qi-Wan (JSSQW) were the most frequent single herb and formula, respectively, prescribed for patients with CKD. The most frequent CHM combination between herbs and formulas was JSSQW, associated with *Rheum officinale* Baill. (RO), SM and *Astragalus membranaceus* (Fisch.) Bunge (AM). The long-term survival rate demonstrated significant benefits for CHM users within a 12-year follow-up period (*P* < 0.004).

**Conclusion:** This nationwide retrospective cohort study provides valuable insight into the characteristics and prescription patterns of CHM usage in CKD patients. JSSQW associated with RO, SM, and AM is the most common CHM prescription. CHM improves long-term survival in patients with CKD, suggesting that CHM is an effective adjuvant therapy for CKD.

## Introduction

Chronic kidney disease (CKD) is a serious issue affecting the health care community globally. Patients with CKD require complicated management protocols, including medicine, lifestyle change, self-care protocol, and possible renal transplant to improve their conditions. Many preventive methods have been explored in order to reduce the burden on health care systems, and lower related medical expenses. As an illustration, current management techniques of diabetic nephropathy (DN), one of the main causes of new onset end-stage renal disease (ESRD) (Li et al., 2017), includes normalization of blood pressure (preferably with renin-angiotensin-aldosterone system antihypertensive agents), strict control of the plasma glucose concentration, lipids lowering, and lifestyle modifications, such as limiting salt and protein intake (Gosmanov et al., 2014). Those measures have been shown to retard the development and progression of DN, however many patients still progress to ESRD (Rosolowsky et al., 2011). Moreover, patients with CKD usually experience diverse symptoms of discomfort or inconvenience due to the disease itself, or treatment that may influence their quality of life or lifespan (Murtagh et al., 2007; Yong et al., 2009). As a result of dissatisfaction with conventional therapy, patients with CKD, even those receiving regular hemodialysis, may choose a second, or adjunct, treatment option for a variety of reasons (Arjuna Rao et al., 2016) including averting CKD progression, resolving concomitant problems such as pruritus, fatigue, depression, anxiety, uremic bruising, and preventing cardiovascular complications (Markell, 2005).

In Asia, and increasingly worldwide, herbal medicines based on traditional Chinese medicine (TCM) theory are commonly applied to prevent CKD progression and ameliorate the side effects resulting from CKD. Chinese herbal medicine (CHM) has a history extending back thousands of years. Its theoretical system, clearly different from conventional medicine, is based on the differentiation of syndromes and patterns. The prescriptions are structured individually according to patients’ constitution and diseases progression. Indeed, the clinical practices of CHM are complicated, and as such cannot be discussed simply in terms of a fixed formula. CHM services are covered by the National Health Care Insurance system (NHCIS) in Taiwan. The database collects information related to the application of CHM in clinical practice. Through analysis of the NHCIS database, we identified the characteristics and prescription patterns of CHM use in patients with CKD, providing insight into the effectiveness of CHM in clinical application and a basis for future research.

## Materials and Methods

### Data Sources

The government of Taiwan has provided the NHCIS to all residents since March 1, 1995. The aim of the NHCIS is to ensure the health of the population of Taiwan by offering medical services to those registered, which covers more than 99% of the population. The National Health Insurance Research Database (NHIRD) is a working database containing the medical care records of the NHCIS, which consist of information regarding patients’ clinical visits, including complete prescription lists, operations undergone, and hospitalization records. For this particular study, we accessed and applied a sub-dataset of the NHIRD, namely the Longitudinal Health Insurance Database 2000 (LHID 2000). The demographic characteristics of patients recovered from the LHID 2000 were tested using the Chi-square test. The identification of patients was transformed into a set of random sequences of letters and numbers.

### Study Population and Variables

**FIGURE 1:**
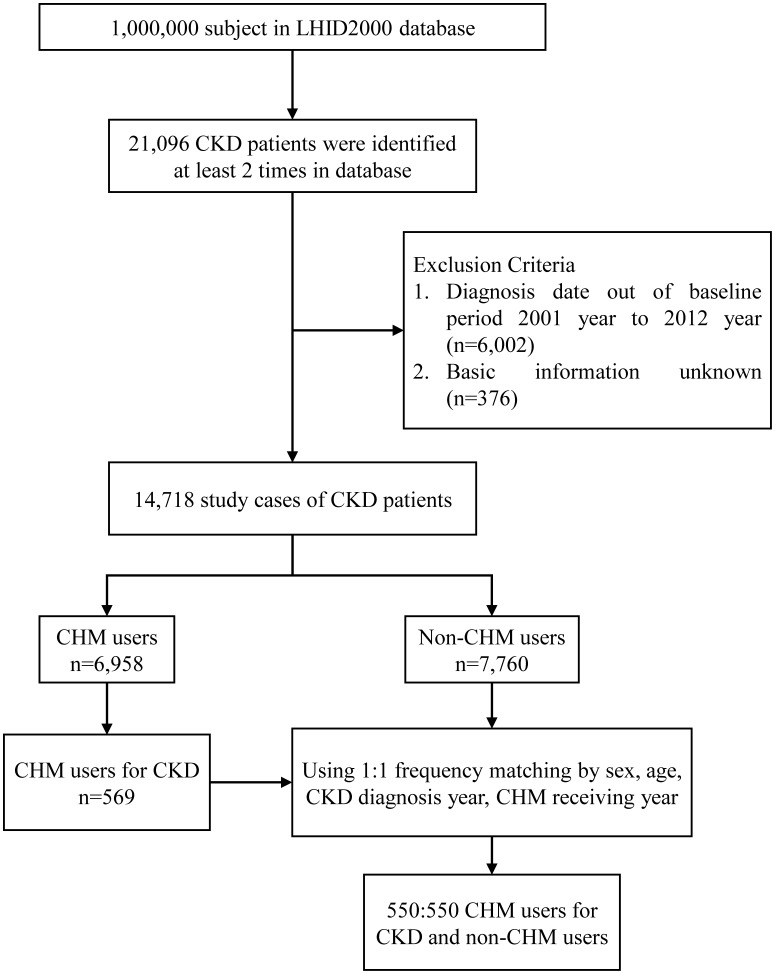
Flow chart of study cases (Chinese herbal medicine, CHM) from Longitudinal Health Insurance Database (LHID2000) in Taiwan during 2001–2012.

The process of subject selection from 1 million individuals in the LHID 2000 is illustrated by flow chart (**Figure 1**). Those patients with CKD were identified by International Classification of Diseases, Ninth Revision, Clinical Modification (ICD-9-CM) code 585, and had to have been diagnosed at least twice before being included in the study population. After screening for the exclusion criteria of diagnostic date and basic information, 14,718 CKD patients were recruited. Patients with CKD, having had at least one record of CHM clinical visit during the study period, were defined as CHM users (*n* = 6,958). Non-CHM users were the CKD population who never recorded a visit to a CHM clinic during the study period (*n* = 7,760). The CHM users included the CKD patients receiving CMH for any reason. The CHM users for CKD were defined to indicate CKD patients receiving CHM for treating CKD. The study collected and analyzed demographic characteristics and claims data of both the CHM user and non-CHM user cohorts (*n* = 550) matched by sex, age, diagnosis date of CKD, and date of CHM clinic visit.

Demographic characteristics including sex, age, job type, residential area, and comorbidity were considered as covariates to adjust the analysis. The study population was divided into four age groups: patients under 20; 20–39; 40–59; and 60 years or older. Job types were classified as office workers, manual workers, and others. The residential areas were divided into four regions: northern Taiwan; central Taiwan; southern Taiwan; eastern Taiwan and offshore islands. The comorbid medical conditions for each individual were evaluated, and certain comorbid diseases were identified, such as diabetes (ICD-9-CM: 250), hypertension (ICD-9-CM: 401-405), ileal conduit (ICD-9-CM: 56.51), neurogenic bladder (ICD-9-CM: 596.54 and 344.61) and urinary tract infection (ICD-9-CM: 599.0, 595.0, and 590.0).

Network analysis of CHM prescriptions comprising herbal formulas and single herbs was analyzed by open-sourced freeware NodeXL^1^ to inspect the core application patterns of CHM for the treatment of CKD patients. Therapeutic actions and indications were recorded based on TCM theory. The two most common herbal combinations were identified and applied to this network analysis. The thicker line width in the illustration defines frequency of connections between formulas and herbs, indicating more significant prescription patterns in the network.

### Ethics Statement

This study was conducted based on the Helsinki Declaration. This study was approved by the Review Board and Ethics Committee of China Medical University Hospital, Taiwan (CMUH104-REC2-115(CR-1)). As all of the datasets were de-identified from LHID 2000, the review board waived the requirement for obtaining informed consent from patients.

### Statistical Analyses

Categorical variables were expressed in counts and percentages, and those were further tested by Chi-square test to evaluate the association between CHM users and non-CHM users in patients with CKD. The mean age between the two groups was tested by two sample Student’s *t*-test. Univariate and multivariable logistic regression was used to assess the preference of CHM use and estimated odds ratios (ORs) with confidence intervals (95% CIs). In multivariable logistic regression, sex, age, job type, residential area, and all comorbidities were considered via adjustment. Network analysis was utilized to analyze the relationship between two Chinese herbal products. The survival analysis applied Kaplan-Meier method to compare the survival rate between CHM users and non-CHM users. Statistical analyses in this study were run by the statistical software package, SAS, version 9.4 (SAS Institute, Inc., Cary, NC, United States) with significant level α = 0.05.

## Results

The results of this study elucidate the data of CHM users and non-CHM users for the treatment of patients with CKD. After matching by sex and age, both the CHM users and non-CHM users cohorts consisted of 550 subjects each. Insignificant association of sex between the two groups is demonstrated in **Table 1** (*p*-value = 0.99). The mean age in both the CHM and non-CHM group was approximately 59 years, and the difference of mean age was not significant (*p*-value = 0.82). Similarly, sex, age, and job type each indicated no special preference for CHM use, nor did area of residence. In a comparison of comorbidities, none were associated with CHM use, except for diabetes (*p*-value < 0.0001), and urinary tract infection (*p*-value = 0.01). Odds ratios of all comorbidities were not significant, except for diabetes (adjusted OR = 0.57, 95% CI = 0.44-0.74, *p*-value < 0.001), and urinary tract infection (adjusted OR = 0.69, 95% CI = 0.49–0.98, *p*-value < 0.05).

**Table 1:** Demographic characteristics and odds ratio with 95% confidence interval estimated by logistic regression of patients diagnosed with CKD in Taiwan during 2001–2012.

Variable	Non-CHM		CHM		*p* value	OR (95% CI)	
	*N* = 550		*N* = 550				
	50.00%		50.00%				
	n	%	n	%		Crude	Adjusted
**Sex**					0.99		
Female	236	42.9	236	42.9		1	Ref
Male	314	57.1	314	57.1		1 (0.79–1.27)	0.9 (0.7–1.16)
**Age at baseline**					0.99		
<20	3	0.55	3	0.55		1	Ref
20–39	38	6.91	38	6.91		1 (0.19–5.27)	0.97 (0.18–5.22)
40–59	240	43.6	240	43.6		1 (0.2–5)	1.01 (0.2–5.19)
≥60	269	48.9	269	48.9		1 (0.2–5)	1.08 (0.21–5.6)
Mean(SD)^‡^	59.5(13.00)		59.3(12.88)		0.82		
**Job type**					0.05		
Office worker	225	40.9	265	48.2		1	Ref
Manual worker	273	49.6	243	44.2		0.76 (0.59–0.97)^∗^	0.78 (0.6–1)
Others	52	9.45	42	7.64		0.69 (0.44–1.07)	0.71 (0.45–1.12)
**Area**					0.04		
Northern Taiwan	219	39.8	207	37.6		1	Ref
Central Taiwan	158	28.7	192	34.9		1.29 (0.97–1.71)	0.99 (0.73–1.35)
Southern Taiwan	139	25.3	132	24		1 (0.74–1.36)	0.99 (0.73–1.35)
Eastern Taiwan and offshore islands	34	6.18	19	3.45		0.59 (0.33–1.07)	0.67 (0.37–1.23)
**Comorbidity**							
Diabetes					<0.001		
No	284	51.6	356	64.7		1	Ref
Yes	266	48.4	194	35.3		0.58 (0.46–0.74)^∗∗∗^	0.57 (0.44–0.74)^∗∗∗^
Hypertension					0.43		
No	118	21.5	129	23.5		1	Ref
Yes	432	78.6	421	76.6		0.89 (0.67–1.18)	1.11 (0.81–1.53)
Ileal conduit					0.32		
No	547	99.5	544	98.9		1	Ref
Yes	3	0.55	6	1.09		2.01 (0.5–8.08)	2.45 (0.59–10.1)
Neurogenic bladder					0.56		
No	545	99.1	543	98.7		1	Ref
Yes	5	0.91	7	1.27		1.41 (0.44–4.45)	1.41 (0.42–4.72)
Urinary tract infection					0.01		
No	447	81.3	478	86.9		1	Ref
Yes	103	18.7	72	13.1		0.65 (0.47–0.91)^∗^	0.69 (0.49–0.98)^∗^

*^∗^p < 0.05, ^∗∗∗^p < 0.001, ^‡^Student’s t-test. CHM, Chinese herbal medicine; SD, standard deviation; OR, odds ratio; CI, confidence interval. The mean (median) of follow-up duration was 1.00 (0.27) years and 3.93 (2.93) for case cohort and compared cohort. Adjusted OR: multiple variable logistic regression adjusted by sex, age, job type, area, and all comorbidities.*

The top five single herbs prescribed for the treatment of CKD patients in CHM outpatient services were *Salvia miltiorrhiza* Bunge (SM), *Rheum officinale* Baill. (RO), *Astragalus membranaceus* (Fisch.) Bunge (AM), seed of *Plantago asiatic* L. (PA), *Eucommia ulmoides* Oliv. (**Table 2**). Of these, SM was the most frequently used single herb, registering 9,376 person-days; followed by RO and AM, with 8,735 and 7,284 person-days, respectively. Among the top five single herbs, AM had the highest average daily dose at 1.5 g. The average duration of prescription for all single herbs was more than 10 days. The ten most common formulas prescribed for the treatment of patients with CKD were shown in **Table 3**. Ji-Sheng-Shen-Qi-Wan (JSSQW) was the most common formula, with 11,347 person-days, an average daily dose of 4.6 g, and an average prescription duration of 11.9 days. The person-days registered for JSSQW were more than twofold higher than those of Liu-Wei-Di-Huang-Wan (LWDHW). Of the top five formulas, Jia-Wei-Xiao-Yao-San (JWXYS) had the highest average daily dose, and highest average duration for a prescription, at approximately 2 weeks. The composition of ten formulas were shown in **Supplementary Table 1**.

**Table 2:** Ten most common single herbs prescribed for patients with CKD.

Prescription name	frequency	Number of person-days	Average daily dose (g)	Average duration for prescription (days)
*Salvia miltiorrhiza* Bunge	737	9376	1.3	12.7
*Rheum officinale* Baill.	669	8735	1	13.1
*Astragalus membranaceus* (Fisch.) Bunge	611	7284	1.5	11.9
*Seed of Plantago asiatica* L.	324	4039	1.4	12.5
*Eucommia ulmoides* Oliv.	289	3450	1	11.9
*Seed of Ziziphus jujuba var. spinosa* (Bunge) Hu ex H.F.Chow	300	3245	1.4	10.8
*Atractylodes macrocephala* Koidz	239	3124	1.2	13.1
*Cistanche deserticola* Ma	227	2698	1.1	11.9
*Wolfiporia extensa*	214	2594	1.5	12.1
*Scutellaria baicalensis* Georgi	196	2555	1.3	13

**Table 3:** Ten most common formula prescribed for patients with ckd.

Prescription name (in Chinese)	frequency	Number of person-days	Average daily dose (g)	Average duration for prescription (days)
Ji-Sheng-Shen-Qi-Wan	955	11347	4.6	11.9
Liu-Wei-Di-Huang-Wan	420	5392	5	12.8
Jia-Wei-Xiao-Yao-San	345	5142	5.1	14.9
Zhu-Ling-Tang	366	4660	4	12.7
Ba-Wei-Di-Huang-Wan	385	4572	4.5	11.9
Gui-Pi-Tang	366	3780	3.8	10.3
Zhi-Bai-Di-Huang-Wan	342	3689	4.1	10.8
Bu-Yang-Huan-Wu-Tang	183	3271	5	17.9
Ma-Zi-Ren-Wan	151	2082	3.3	13.8
Zhi-Gan-Cao-Tang	173	2028	4	11.7

**FIGURE 2:**
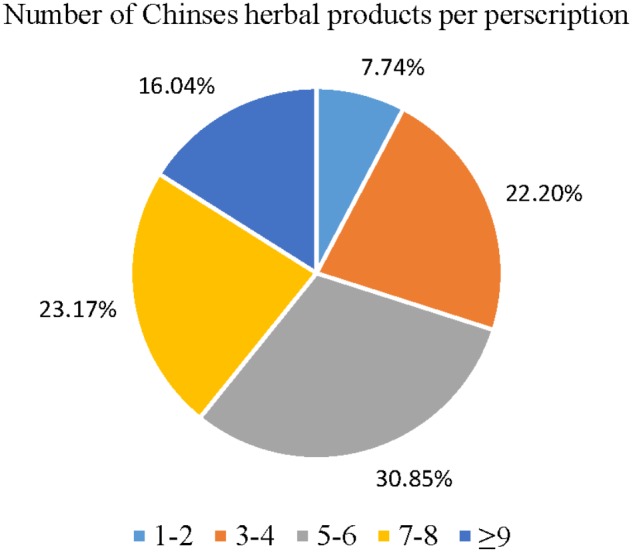
Distribution of Chinese herbal products’ combination in one treatment for patients with CKD.

**FIGURE 3:**
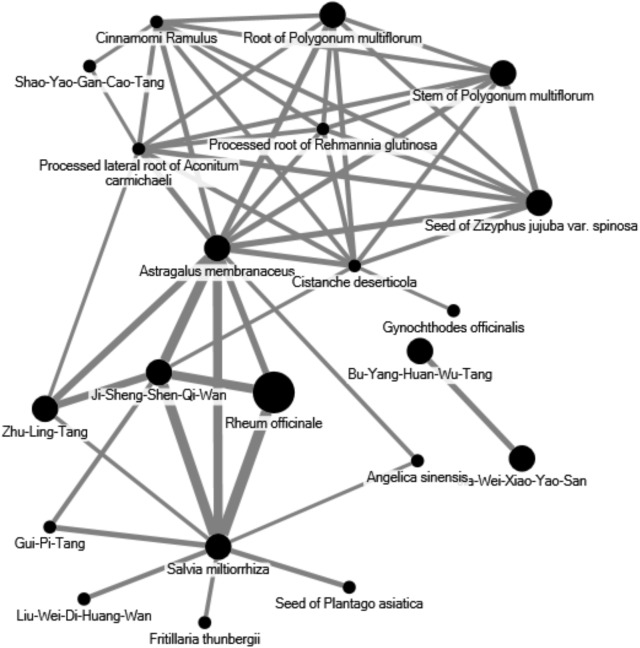
Network analyses of the most frequent 50 herbs and formulas combination for patients with CKD.

The data reveals that 30.85% of prescriptions for CKD patients by CHM practitioners contained five or six compositions per treatment. Prescriptions consisting of three or four, and seven or eight compositions per treatment each accounted for nearly one-quarter of all prescriptions. Interestingly, the data reveals that CHM practitioners seldom prescribed treatments for CKD consisting of one or two compositions (7.74%) (**Figure 2**). The most frequent CHM combination between herbs and formulas was RO associated with SM, AM, and JSSQW (**Figure 3**). AM was the most popular CHM combined with eleven different herbs and formulas. In clinical practice, JSSQW was the most commonly prescribed formula, associated with the top three single herbs of SM, AM, and RO.

**FIGURE 4:**
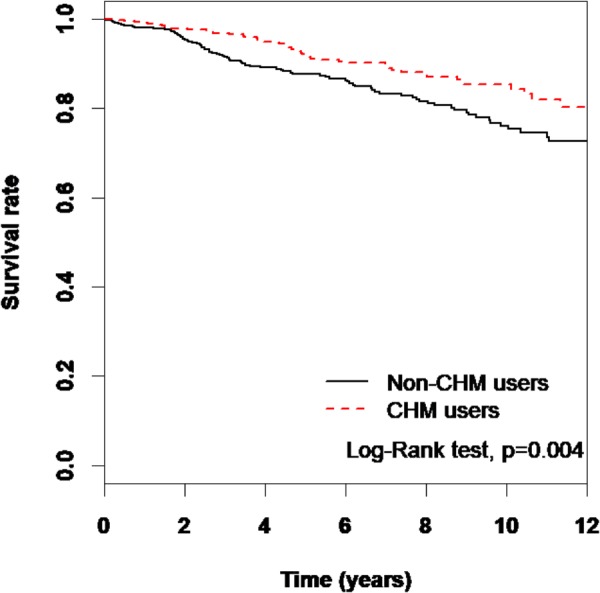
The estimated survival rate between the CHM users and the non-CHM users by Kaplan-Meier analysis.

Analysis of the data concerning CKD patients reveals notable evidence regarding the benefits of using CHM. The long-term survival rate illustrated by the data displayed significant differences between CHM users and non-CHM users (**Table 4**). The *p* value of the log-rank test was less than 0.004 within 12 years of follow-up (**Figure 4**). Besides, the *Salvia miltiorrhiza* Bunge (aHR = 0.4, 95% CI = 0.17-0.95, *p*-value = 0.04) and Liu-Wei-Di-Huang-Wan (aHR = 0.34, 95% CI = 0.12-0.95, *p*-value = 0.04) might have the effect to reduce mortality risk in CKD patients with CHM (**Supplementary Table 2**).

**Table 4:** Survival rate in different follow-up durations between non-CHM and CHM patients.

Follow-up duration (years)	Survival rate (%)		*p* value
	Non-CHM	CHM	
≤2	95.4	97.9	0.03
≤4	89.1	95	0.001
≤8	81.6	87.5	0.008
≤12	72.6	80.3	< 0.001

## Discussion

CHM is the most common form of both alternative and complementary medicine in Asia. As such, patients often turn to CHM to manage health complications when they are not satisfied with conventional therapy, and CKD patients are no exception. However, recent studies have indicated that some Chinese herbs which contain aristolochic acid (AA) can be associated with renal damage and urinary tract cancer. Therefore, some doubts have been raised as to the suitability of using integrative CHMs for CKD patients (Lai et al., 2009; Bara et al., 2017). As CHM prescriptions are included in Taiwan’s NHCIS, the database provides valuable long-term data to analyze the effectiveness of CHM as prescribed by licensed CHM practitioners to CKD patients. According to a previous NHCIS database study, CKD patients who received non-aristolochic acid CHM showed improved survival rates during a 9-year follow-up period (Hsieh et al., 2014). Another such study showed lower cumulative incidence of end-stage renal disease within 6 years of follow-up (Lin M.Y. et al., 2015). Similarly, the present study reveals that CHM users demonstrate a significantly improved survival rate compared to non-CHM users during a 12-year follow-up period. The results of this, and similar previous studies of the Taiwanese population, indicate that there are significant benefits to using CHM as prescribed by licensed CHM practitioners to CKD patients.

The analysis of patient demographic characteristics showed that none of sex, age, job type, or residential area indicated special preference to CHM usage in patients with CKD. Furthermore, comorbidities associated with hypertension, ileal conduit, and neurogenic bladder showed no significant differences between the two cohorts. However, diabetes and urinary tract infection showed lower ratios in the CHM group, indicating that CHM might play an essential role in reducing the risk of renal deterioration in type 2 diabetes, as well as urinary tract infection (Hsu et al., 2014). Some other prescriptions identified in the network analyses, such as Zhu-Ling-Tang and seed of *Plantago Asiatic*, are commonly used in CHM clinical practice to manage urinary tract infection; however, the therapeutic functions, and specific mechanisms related to CKD remain unclear. Whether the difference identified in the data is due to the protection provided by CHM management warrants further investigation.

The network analyses revealed that JSSQW was the most popular formula prescribed in clinical practice. JSSQW is a derivative formula of LWDHW. In fact, LWDHW, the 4th of the 10 most commonly prescribed formulas, and its derivative formulas, are frequently prescribed for patients with CKD. In addition to JSSQW, Ba-Wei-Di-Huang-Wan (BWDHW) and Zhi-Bai-Di-Huang-Wan (ZBDHW) are formulas both derived from LWDHW. Thus, LWDHW should in effect be considered the most popular formula prescribed for CKD patients. According to TCM theory, LWDHW is the fundamental formula to tonify the kidneys. Based on TCM concepts, practitioners can adjust the constitution of each individual by tonifying the kidney Yang with BWDHW, or nourishing the kidney Yin with ZBDHW. As CKD patients often suffer from general weakness, fatigue, and edema, JQSSW has been shown effective at relieving such symptoms and signs. As reported, LWDHW has demonstrated the inhibitory effect of smoothing muscle cell contractility *in vitro*, and reducing the NF-κB expression in kidney lesion rat models (Hao et al., 2010; Lin Y.J. et al., 2015). Moreover, LWDHW has also been reported as the most frequent formula prescribed for non-insulin dependent type 2 diabetes patients (Lee et al., 2016). Population-based studies have also shown that LWDHW is one of the most common CMHs prescribed to reduce the risk of kidney failure and improve the overall survival rate of individuals with hypertension among type 2 diabetes patients (Hsu et al., 2014; Lin Y.J. et al., 2015). However, the effect of LWDHW on reducing kidney failure was not directly correlated to dosage. It is important to note that there was no difference in the risk of developing kidney failure between LWDHW users and non-LWDHW users (Hsu et al., 2014); furthermore, the exact renal protection mechanism of LWDHW remains unclear.

The three most frequently used single herbs, *Salvia miltiorrhiza* Bunge (SM), *Astragalus membranaceus* (Fisch.) Bunge (AM) and *Rheum officinale* Baill. (RO), when joined with JSSQW are all considered as renoprotective CHM (Wojcikowski et al., 2006; Tong et al., 2010). According to TCM theory, SM promotes blood flow to resolve stasis and is usually adopted to treat diseases related to circulatory obstruction. Extract from SM has been demonstrated to exhibit renal protective effects in an adenine-induced renal failure rat animal model study (Chung et al., 1988). It has also been shown that several compounds, isolated from SM, suppressed the generation and release of nitric oxide and reactive oxygen species to ameliorate renal failure (Yokozawa and Chen, 2000). In addition, the magnesium lithospermate B isolated from SM had inhibitory effects on urine protein excretion, blood urine nitrogen, mesangial proliferation, tubule-interstitial lesion, and glomerular sclerotic change in nephrectomized rats (Yokozawa et al., 1995). Another major compound, tanshinone IIA, inhibited over-activity of glycogen synthase kinase 3β to protect transition of kidney injury from acute to chronic changes (Jiang et al., 2016). AM is one of the most important herbs to tonify Qi. CKD patients are usually complicated with edema, so AM is usually applied for tonifying Qi in order to drain dampness. Several studies have demonstrated that the renal protective effectiveness of AM included increased creatinine, serum albumin, and hemoglobin clearance, as well as decreased blood pressure and proteinuria (Zhang et al., 2014). Astragaloside IV, derived from AM, can decrease proteinuria excretion of membranous nephropathy. The possible mechanism functions by restoring podocyte morphology and cytoskeleton loss through the mitogen-activated protein kinase pathway by reducing phosphorylation of JNK and ERK1/2 (Zheng et al., 2012). Another report has also verified that astragaloside IV reduced proteinuria in diabetic nephropathy in an animal model study through inhibition of endoplasmic reticulum stress (Wang et al., 2015). The main function of RO in TCM theory is as a potent purgative to free the bowels and remove excessive heat, stagnant matter, and toxic substances, as well as promote blood flow to resolve stasis. RO is considered a herb effective at eliminating metabolic waste for CKD patients. As reported, RO administration in an adenine-induced rat CKD model disclosed the positive effects of lowering serum creatinine and blood urine nitrogen (Yokozawa et al., 1984). In addition to renal function, RO also reduced proteinuria and the severity of glomerulosclerosis in a nephrectomy CKD rat model (Zhang and el Nahas, 1996). A randomized clinical trial showed that the addition of RO as a supplement associated with telmisartan in stage 3 and 4 CKD patients for 12 weeks indicated marked improvement in clinical features and in biochemical parameters compared to conventional therapy alone (Khan et al., 2014). Further, rhein, an anthraquinone compound isolated from RO, provided renal protective properties by reversing Klotho repression via promoter demethylation in an adenine-induced mouse model (Zhang et al., 2017). The biological activities associated with experimental models of extracted ingredients in herbal formulas and single herbs mentioned above were summarized as shown in **Supplementary Table 3**.

In Taiwan, prescriptions for CKD patients usually combined several CHMs. In our study, the combination of 3 to 8 CHMs is the most common prescription for patients, so we used network analyses to examine the various associations and interactions among the drugs. RO combined with SM indicated purging methodology to remove the accumulation of metabolic waste in patients with CKD. The combination of rhein in RO and danshensu in SM has been reported to exert a synergetic effect to improve renal function, increase blood supply to the kidneys, and prevent fibrotic changes by anti-inflammation and anti-apoptosis (Guan et al., 2015). TCM practitioners also adopted a tonifying methodology to repair tissue damage or organ dysfunction in CKD patients. The combination of AM, *Angelica sinensis* (Oliv.) Diels (AS), *Polygonum multiflorum* Thunb., and JSSQW was frequently applied as invigorating therapy. Wang et al has reported that AM combined with AS in a puromycin-induced nephrosis rat model study inhibited the fibortic change that is similar to enalapril, but not through renin-angiotensin-aldosterone machinery (Wang et al., 2004). The major symptoms and signs, including renal dysfunction and urine protein loss, usually affect the individual constitution as both essence deficiency and waste accumulation in patients with CKD, based on TCM theory, so that the two-pronged methodology of tonifying and elimination are frequently applied in clinical practice. Bu-Yang-Huan-Wu-Tang (BYHWT), composed of AM, AS, and four other herbs with association to promoting blood circulation and freeing blood stasis, is a standard formula with the functional effects of both nourishing the Qi and eliminating stasis. BYHWT has been noted as the most commonly prescribed CHM for stroke (Chang et al., 2016), while also being reported to improve the frequency and severity of seizures in patients with epilepsy refractory to standard antiepileptic medications (Hijikata et al., 2006).

This study obtained data from the sub-dataset of the NHIRD, with an extremely high coverage rate of the Taiwanese population and healthcare institutions, which ensures this study has minimal selection bias of both CHM and non-CHM cohorts among CKD patients. We analyzed the long-term treatment effects by comparing the different survival rates of CKD patients between CHM and non-CHM users. However, we did not have access to precise patient physical examinations, laboratory data (blood urine nitrogen, serum creatinine, electrolytes, albumin, hemoglobin, and urine protein, etc.), or lifestyle details, which were not included in the NHIRD. Therefore, we didn’t have access to the exact clinical condition of patients in order to compare the severity of CKD. We also could not clarify if CHM-users tended to maintain healthier lifestyles, which could influence the survival rate of CKD patients. Nevertheless, this large-scale population-based retrospective cohort analysis provides valuable information on the effectiveness of CHM combination therapy in CKD patients to improve the long-term survival rate.

## Conclusion

Altogether, our results indicate that patients with CKD using CHM treatments exhibited an increased long-term survival rate within a 12-year follow-up period. This study provides evidence supporting the use of CHM as an adjuvant therapeutic option for patients with advanced kidney impairment. This population-based retrospective study discloses the characteristics and specific usage patterns of TCM prescriptions for patients with CKD in clinical application. However, further prospective cohort study, and longitudinal evaluations for CHM use in patients with CKD are necessary.

## Author Contributions

K-CH wrote the manuscript and interpreted the data. Y-CS collected, assembled and analyzed the data. M-FS provided study materials. S-TH designed, conceived the study and amended the manuscript. K-CH, Y-CS, M-FS, and S-TH approved the final manuscript.

## Conflict of Interest Statement

The authors declare that the research was conducted in the absence of any commercial or financial relationships that could be construed as a potential conflict of interest.

## Footnotes

1 http://nodexl.codeplex.com/

## Abbreviations

AA: aristolochic acid
AM: Astragalus membranaceus (Fisch.) Bunge
AS: Angelica sinensis (Oliv.) Diels
BWDHW: Ba-Wei-Di-Huang-Wan
BYHWT: Bu-Yang-Huan-Wu-Tang
CHM: Chinese herbal medicine
CI: confidence interval
CKD: chronic kidney disease
DN: diabetic nephropathy
ESRD: end-stage renal disease
ICD-9-CM: International Classification of Diseases, Ninth Revision, Clinical Modification
JSSQW: Ji-Sheng-Shen-Qi-Wan
JWXYS: Jia-Wei-Xiao-Yao-San
LHID 2000: Longitudinal Health Insurance Database 2000
LWDHW: Liu-Wei-Di-Huang-Wan
NHCIS: National Health Care Insurance System
NHIRD: National Health Insurance Research Database
OR: odds ratio
PA: Plantago Asiatic L
RO: Rheum officinale Baill
SM: Salvia miltiorrhiza Bunge
TCM: traditional Chinese medicine
ZBDHW: Zhi-Bai-Di-Huang-Wan.
