# Supplementary material for: Chinese Herbal Medicine Improves the Long-Term Survival Rate of Patients With Chronic Kidney Disease in Taiwan: A Nationwide Retrospective Population-Based Cohort Study
Source: Front Pharmacol. 2018 Oct 1;9:1117. doi: 10.3389/fphar.2018.01117 (PMC6174207; doi:10.3389/fphar.2018.01117)
Supplement: Supplementary file 1 [file Table_1.DOCX]

Supplementary Table 1. The composition of ten most common herbs formulas prescribed for chronic kidney disease

| Ten formulas | Composition |
| --- | --- |
| Ji-Sheng-Shen-Qi-Wan | *Rehmannia glutinosa (Gaertn.) DC., Cornus officinalis Siebold & Zucc., Dioscorea oppositifolia L., Wolfiporia extensa, Paeonia × suffruticosa Andrews, Alisma orientale (Sam.) Juz., Cinnamomum cassia (L.) J.Presl, Aconitum carmichaeli Debeaux, Achyranthes bidentata Blume, seed of Plantago asiatica L.* |
| Liu-Wei-Di-Huang-Wan | *Rehmannia glutinosa (Gaertn.) DC., Cornus officinalis Siebold & Zucc., Dioscorea oppositifolia L., Wolfiporia extensa, Paeonia × suffruticosa Andrews, Alisma orientale (Sam.) Juz.* |
| Jia-Wei-Xiao-Yao-San | *Angelica sinensis (Oliv.) Diels, Wolfiporia extensa, Gardenia jasminoides J.Ellis, Mentha arvensis L., Paeonia lactiflora Pall., Bupleurum chinense DC., Glycyrrhiza uralensis Fisch., Atractylodes macrocephala Koidz, Paeonia × suffruticosa Andrews, Zingiber officinale Roscoe* |
| Zhu-Ling-Tang | *Polyporus umbellatus, Wolfiporia extensa, Alisma orientale (Sam.) Juz., gelatin of Equus asinus, talc* |
| Ba-Wei-Di-Huang-Wan | *Rehmannia glutinosa (Gaertn.) DC., Cornus officinalis Siebold & Zucc., Dioscorea oppositifolia L., Wolfiporia extensa, Paeonia × suffruticosa Andrews, Alisma orientale (Sam.) Juz., Cinnamomum cassia (L.) J.Presl, Aconitum carmichaeli Debeaux* |
| Gui-Pi-Tang | *Panax ginseng C.A.Mey., Astragalus membranaceus (Fisch.) Bunge, Atractylodes macrocephala Koidz, Wolfiporia extensa, Angelica sinensis (Oliv.) Diels, seed of Ziziphus jujuba var. spinosa (Bunge) Hu ex H.F.Chow, Dimocarpus longan Lour., Polygala tenuifolia Willd., Aucklandia costus Falc., Glycyrrhiza uralensis Fisch., Zingiber officinale Roscoe, Ziziphus jujuba Mill.* |
| Zhi-Bai-Di-Huang-Wan | *Rehmannia glutinosa (Gaertn.) DC., Cornus officinalis Siebold & Zucc., Dioscorea oppositifolia L., Wolfiporia extensa, Paeonia × suffruticosa Andrews, Alisma orientale (Sam.) Juz., Anemarrhena asphodeloides Bunge, Phellodendron chinense C.K.Schneid.* |
| Bu-Yang-Huan-Wu-Tang | *Astragalus membranaceus (Fisch.) Bunge, Angelica sinensis (Oliv.) Diels, Paeonia lactiflora Pall., Pheretima aspergillum, Ligusticum striatum DC., Prunus persica (L.) Batsch, Carthamus tinctorius L.* |
| Ma-Zi-Ren-Wan | *Seed of Cannabis sativa L., Paeonia lactiflora Pall., Citrus × aurantium L., Rheum officinale Baill., Magnolia officinalis Rehder & E.H.Wilson, seed of Prunus armeniaca L.* |
| Zhi-Gan-Cao-Tang | *Glycyrrhiza uralensis Fisch., Panax ginseng C.A.Mey., Twig of Cinnamomum cassia (L.) J.Presl, Zingiber officinale Roscoe, gelatin of Equus asinus, Rehmannia glutinosa (Gaertn.) DC., Ophiopogon japonicus (Thunb.) Ker Gawl., seed of Cannabis sativa L., Ziziphus jujuba Mill.* |
