# Supplementary material for: Chinese Herbal Medicine Improves the Long-Term Survival Rate of Patients With Chronic Kidney Disease in Taiwan: A Nationwide Retrospective Population-Based Cohort Study
Source: Front Pharmacol. 2018 Oct 1;9:1117. doi: 10.3389/fphar.2018.01117 (PMC6174207; doi:10.3389/fphar.2018.01117)
Supplement: Supplementary file 2 [file Table_2.DOCX]

| **Supplementary Table 2. The mortality risk of the commonly used Chinese medicine single herb and formulae in CKD patients with CHM** | | | |
| --- | --- | --- | --- |
| **Single herb** | **Adjusted HR** | **95% CI** | **p value** |
| *Salvia miltiorrhiza* | 0.4 | (0.17-0.95) | 0.04 |
| *Rheum rhabarbarum* | 0.97 | (0.49-1.91) | 0.93 |
| Formula | Adjusted HR | 95% CI | p value |
| Ji-Sheng-Shen-Qi-Wan | 0.62 | (0.32-1.2) | 0.16 |
| Liu-Wei-Di-Huang-Wan | 0.34 | (0.12-0.95) | 0.04 |
| CI=confidence interval; HR=hazard ratio; Adjusted: model adjusted sex, age, job type, area and all comorbidities. | | | |
