# Supplementary material for: Chinese Herbal Medicine Improves the Long-Term Survival Rate of Patients With Chronic Kidney Disease in Taiwan: A Nationwide Retrospective Population-Based Cohort Study
Source: Front Pharmacol. 2018 Oct 1;9:1117. doi: 10.3389/fphar.2018.01117 (PMC6174207; doi:10.3389/fphar.2018.01117)
Supplement: Supplementary file 3 [file Table_3.DOCX]

**Supplementary Table 3. The biological activities associated with experimental models of extracted ingredients in herbal formulas and single herbs**

| **Herbal formulas/ single herbs** | **Form** | **Biological effects and related molecular mechanisms** | **Models** |
| --- | --- | --- | --- |
| Liu-Wei-Di-Huang-Wan | Pill | inhibit the expression of NF-kappaB protein to alleviate the renal oxidative lesion | Rat ([Hao et al., 2010](#_ENREF_7)) |
|  | water extract | reduce myosin light chain phosphorylation to inhibit smooth muscle cell contractility | Rat aortic smooth muscle cells (A10) (Lin, Y.J. et al., 2015). |
| *Salvia miltiorrhiza* | extract | suppress the generation and release of nitric oxide and scavenge reactive oxygen species | Mice (Yokozawa and Chen, 2000) |
|  | magnesium lithospermate B | inhibited urine proteins excretion, blood urine nitrogen, mesangial proliferation, tubule-interstitial lesion and glomerular sclerotic lesions | Rat (Yokozawa et al., 1995) |
|  | Tanshinone IIA | inhibited glycogen synthase kinase 3β overactivity | Mice (Jiang et al., 2016) |
| *Astragalus membranaceus* | Astragaloside IV | restored podocyte morphology and cytoskeleton loss by reduce phosphorylation of JNK and ERK1/2 | Mouse podocyte cell (MPC5) (Zheng et al., 2012) |
|  | Astragaloside IV | prevented the phosphorylation of eIF2α, PERK and JNK, and inhibited the expression of GRP78 and ORP150 | Rat (Wang et al., 2015) |
| *Rheum officinale* | Rhein | reversing Klotho promoter hypermethylation | Mice (Zhang et al., 2017) |
| *Rheum officinale* and *Salvia miltiorrhiza* | rhein and danshensu | anti-inflammation by down-regulation of NF-κB related pathway; anti-fibrosis by down-regulation of TGF-β/Smad3 pathway; inhibition of apoptosis by up-regulating Bcl-2 and down-regulating Bax | Rat and HK-2 cell (Guan et al., 2015) |
| *Astragalus membranaceus* and *Angelica sinensis* | extract | reduce the number of ED-1-positive, and a-actin positive cells and expression of osteopontin | Rat (Wang et al., 2004) |
